# Elemental Analysis of Bone, Teeth, Horn and Antler in Different Animal Species Using Non-Invasive Handheld X-Ray Fluorescence

**DOI:** 10.1371/journal.pone.0155458

**Published:** 2016-05-19

**Authors:** Kittisak Buddhachat, Sarisa Klinhom, Puntita Siengdee, Janine L. Brown, Raksiri Nomsiri, Patcharaporn Kaewmong, Chatchote Thitaram, Pasuk Mahakkanukrauh, Korakot Nganvongpanit

**Affiliations:** 1 Animal Bone and Joint Research Laboratory, Department of Veterinary Biosciences and Public Health, Faculty of Veterinary Medicine, Chiang Mai University, Chiang Mai 50100, Thailand; 2 Elephant Research and Education Center, Faculty of Veterinary Medicine, Chiang Mai University, Chiang Mai 50100, Thailand; 3 Smithsonian Conservation Biology Institute, Center for Species Survival, National Zoological Park 1500 Remount Road, Front Royal, Virginia, 22630, United States of America; 4 Veterinary Conservation and Research Section, Chiang Mai Night Safari, Chiang Mai 50100, Thailand; 5 Phuket Marine Biological Center, Phuket 83000, Thailand; 6 Excellence Center in Osteology Research and Training Center, Chiang Mai University, Chiang Mai 50200, Thailand; Waseda University, JAPAN

## Abstract

Mineralized tissues accumulate elements that play crucial roles in animal health. Although elemental content of bone, blood and teeth of human and some animal species have been characterized, data for many others are lacking, as well as species comparisons. Here we describe the distribution of elements in horn (Bovidae), antler (Cervidae), teeth and bone (humerus) across a number of species determined by handheld X-ray fluorescence (XRF) to better understand differences and potential biological relevance. A difference in elemental profiles between horns and antlers was observed, possibly due to the outer layer of horns being comprised of keratin, whereas antlers are true bone. Species differences in tissue elemental content may be intrinsic, but also related to feeding habits that contribute to mineral accumulation, particularly for toxic heavy metals. One significant finding was a higher level of iron (Fe) in the humerus bone of elephants compared to other species. This may be an adaptation of the hematopoietic system by distributing Fe throughout the bone rather than the marrow, as elephant humerus lacks a marrow cavity. We also conducted discriminant analysis and found XRF was capable of distinguishing samples from different species, with humerus bone being the best source for species discrimination. For example, we found a 79.2% correct prediction and success rate of 80% for classification between human and non-human humerus bone. These findings show that handheld XRF can serve as an effective tool for the biological study of elemental composition in mineralized tissue samples and may have a forensic application.

## Introduction

Mineralized tissues, such as bone, teeth, antler and horn, are important elemental storage sites in animals. These tissues contain necessary elements, both major, such as calcium (Ca), phosphorus (P), magnesium (Mg) and sulphur (S), and trace elements, such as iron (Fe), zinc (Zn), manganese (Mn) and cadmium (Cd). Most elemental research has focused on the major elements, especially Ca, P, Mg, due to their crucial role in bone metabolism [1]. However, other elemental evaluations and comparisons across tissue types and species are required to more fully understand their biological function.

Investigations of elemental distribution and accumulation in tissues contribute to studies of physiology, ecology, environmental contamination and forensic science. For example, decreases in major and/or trace elements are related to pathogenesis of abnormal tissues, such as osteoarthrosis and bone fractures in humans [2] and osteoarthritis in dogs [3]. Elemental analyses also can indicate dietary habits and environmental influences on mineral accumulation, and the relative contribution of food sources to the diet [4]. From an environmental standpoint, global monitoring of toxic heavy metals, such as lead (Pb), arsenic (As), Cd and mercury (Hg) is crucial to verify deleterious effects on aquatic animal species [5–8]. Last, elemental profiling can be used as a tool by law enforcement agencies to identify species and animal origins, particularly as it pertains to endangered wildlife [3, 9, 10].

X-ray fluorescence (XRF) is used for routine, relatively non-destructive chemical analyses of rocks, minerals, sediments and fluids, and can provide important information on the elemental components of various biological sample types, such as bone [11–14], teeth [3, 12, 15] and antler [16], and forensically to identify the species of body remains [11, 12, 17]. Recent studies have examined lead levels in human bone (*in vivo*) and arsenic levels in human nail clippings by portable XRF [18, 19]. In addition, handheld XRF has been used to sort human from other species’ bone samples with high accuracy [20]. We recently determined the elemental composition of Asian elephant teeth, with subsequent comparisons between 15 other species. Based on discriminate analyses, XRF was able to distinguish between dog, pig, goat, tapir, monkey, and elephant tooth samples with a 100% success rate [3]. Subsequently, we identified differences in elemental composition of human bones between male and female samples: eight (silicon (Si), S, Ca, Mn, Fe, Zn, silver (Ag) and Pb), nine (S, Ca, Fe, zirconium (Zr), Ag, Cd, tin (Sn), antimony (Sb) and Pb) and 10 (P, S, (titanium) Ti, Fe, Zn, Ag, Cd, Sn, Sb and Pb) elements differed by sex for cranium, humerus and os coxae bones, respectively [14]. The accuracy rate for sex estimation by XRF was only ~60–67%, however, so more refining of the technique is needed to make it more reliable for human bones.

The primary aim of this study was to explore in additional mammalian species the distribution of accumulated elements in four dense connective tissue types (horn, antler, teeth, bone) by non-invasive XRF. The secondary aim was to compare results across species to further understand differences in biology and the ability of XRF to discriminate between sources of tissues and tissue types.

## Materials and Methods

### Samples

Animal antler, horn, bone and teeth sample (S1 File) were obtained from the Animal Anatomy Museum, Department of Veterinary Biosciences and Public Health, Faculty of Veterinary Medicine, Chiang Mai University, Chiang Mai, Thailand. Human bone and teeth samples (dry bone) were obtained from donations as cadavers to Department of Anatomy, Faculty of Medicine, Chiang Mai University, Thailand. To use these skeletons, consent was waived by Human Ethics Committee, Faculty of Medicine, Chiang Mai University, Thailand on 2015, and the samples were also anonymized in our study. The use of animal bones from the Animal Anatomy Museum did not require approval by the Animal Ethic Committee, Faculty of Veterinary Medicine, Chiang Mai University.

Samples were dry, maintained at room temperature, and were not stored longer than 12 years after death. They were immediately cleaned upon death, but were not otherwise manipulated (burned or buried), except the elephant skeleton, which was buried for 2 years to decay the soft tissue. None of the samples exhibited pathological lesions or disease conditions.

### X-ray fluorescence measurement

Bone elemental analyses were conducted using a handheld XRF (DELTA Premium, Olympus, USA), which uses a silicon drift detector, detecting from magnesium (12 Mg) through bismuth (83 Bi) on the periodic table. The collimator size was set at 0.3 mm for the analysis-area diameter, and used the standard, mining plus mode. Calibrations were performed before the first use of the handheld XRF for sample analysis each day. Light elements (LE) were those with an atomic number lower than Mg (H^1^-Na^11^), which could not be differentiated as separate elements. For each scan (2 min each), the XRF unit was secured in a stand and the sample was placed directly adjacent to the puncture resistant window of the machine to limit the distance between the detector and samples. Each element was expressed as a percentage obtained from the area under the peak of each element divided by total area for all elements recorded in the scan. Elemental values represent a relative amount (elemental fingerprint), but not actual concentrations of each element in a tested sample. The XRF method was noninvasive, and samples were not manipulated or destroyed in the process of scanning.

### Study design

XRF was used to examine and compare the elemental composition of: 1) antler and horn in 12 mammalian species; 2) teeth from six mammalians and one reptile species; and 3) humerus bones of 14 mammalian species.

#### Study 1: Elemental analysis of horn/antler in 10 species of Bovidae and two species of Cervidae

Skull samples of Bovidae included Asiatic buffalo (*Bubalus bubalis*; n = 3), Barbary sheep (*Ammotragus lervia*; n = 1), domestic goat (*Capra hircus*; n = 3), eland (*Tragelaphus oryx*; n = 1), gemsbuck (*Oryx gazella*; n = 1), Grant's gazelle (*Nanger granti*; n = 2), greater kudu (*Tragelaphus strepsiceros*; n = 1), nyala (*Tragelaphus angasii*; n = 1), red lechwe (*Kobus leche*; n = 1) and sitatunga (*Tragelaphus spekii*; n = 1). Cervidae samples were from spotted deer (*Axis axis*; n = 2) and Sunda sambar (*Rusa timorensis*; n = 2). We scanned six locations on each horn/antler (two scans each; distal, middle and proximal). In addition, the frontal bone of the skulls of spotted deer and Sunda sambar deer were scanned to compare with antler.

#### Study 2: Elemental analysis of teeth from multiple species

Teeth from eight species (six land mammals, one marine mammal and one reptile) were analyzed: deer (*Odocoileus virginianus*, n = 3); dog (*Canis lupus familiaris*, n = 5); elephant (Asian-; *Elephas maximus*, n = 2); horse (*Equus ferus caballus*, n = 3); human (*Homo sapiens*, n = 5); monkey (Assam macaques; *Macaca assamensis*, n = 5); dolphins (Spinner-; *Stenella longirostris*, n = 2); and crocodile (*Crocodylus siamensis*, n = 2). Three teeth per species were scanned by XRF. Molars were evaluated in all mammalian species.

#### Study 3: Elemental analysis of humerus bone in 14 species

Elemental composition of the humerus bone was determined in 14 species: buffalo (Asiatic-, *Bubalus bubalis*; n = 6), cat (*Felis catus*; n = 8), dog (*Canis lupus familiaris*; n = 10), dolphin (Spinner-, *Stenella longirostris*; n = 4), elephant (Asian-, *Elephas maximus*; n = 6), horse (*Equus ferus caballus*; n = 6), human (*Homo sapiens*; n = 10), hyena (*Hyaena hyaena*; n = 4), lion (*Panthera leo*; n = 2), malayan tapir (*Tapirus indicus*; n = 2), Monkey (Assam Macaques, *Macaca assamensis*; n = 10), pig (*Sus scrofa domesticus*; n = 6), sheep (*Ovis aries*; n = 6), tiger (*Panthera tigris*; n = 2). Human, dog, cat, Asiatic buffalo, sheep and lion bone were collected from males, while the remaining samples were of unknown sex origin. Eight location sites on each humerus were scanned.

Additional analyses were conducted on the teeth of six of the aforementioned species (at least three molar teeth per species) for comparison with corresponding bone data: dog (*Canis lupus familiaris*), elephant (Asian; *Elephas maximus*), horse (*Equus ferus caballus*), human (*Homo sapiens*), monkey (Assam macaques; *Macaca assamensis*) and spinner dolphins (*Stenella longirostris*).

### Statistical analyses

Data are presented as mean ± SD. Differences elemental percentages among animals within the Bovidae (10 species) and Cervidae (2 species) families were determined using student’s T-tests. Differences between species for each element in horn, humerus and teeth were tested by one-way ANOVA followed by Tukey’s post hoc test. Differences in elemental percentages between antler and frontal bone were analyzed using T-tests. To compare elements between teeth and humerus in six animal species (elephant, monkey, dog, horse, human and dolphin), t-tests were used and P value < 0.05 was consider significantly difference. Additionally, the ratio of Ca to P in humerus and teeth of six animal species was calculated and tested using student’s T-test between humerus and teeth of each species. The elemental content across animal species in each study was performed by a stepwise discriminant analysis with leave one out classification for species predication.

## Results

### Study 1: Elemental analysis of horn/antler in 10 species of Bovidae and two species of Cervidae

The elemental comparison between Bovidae and Cervidae is outlined in Table 1, and highlights differences in 14 of the 18 elements and LE (P < 0.01) between groups. Chlorine (Cl) was present in horn, but not antler, while vanadium (V), Chromium (Cr), Zr, Ag, Cd, Sn and Sb were found only in antler. Other elements were present in both tissues at differing concentrations: antler had significantly higher P, potassium (K), Ca, Ti, Mn and Fe than horn, while horn presented a significantly higher percentage of S than antler.

**Table 1:** Mean (± standard deviation (SD)) elemental percentages in horn of 10 Bovidae and antler of two Cervidae speces. Data were combined across species within family category.

Specimen	Al	Si	P	S	Cl	K	Ca	Ti	V	Cr	Mn	Fe	Zn	Zr	Ag	Cd	Sn	Sb	LE
**Horn (Bovidae)**	0.711±0.708	1.901±1.691	0.132±0.114	1.924±0.726	2.725±1.833	0.433±0.269	0.505±0.259	0.015±0.006	0	0	0.008±0.008	0.050±0.055	0.020±0.010	0	0	0	0	0	92.050±2.175
**Antler (Cervidae)**	0.980±0.457	3.441±3.101	5.941±1.634	0.300±0.144	0	0.787±0.590	17.4382.585	0.067±0.031	0.018±0.006	0.009±0.001	0.031±0.011	0.124±0.099	0.023±0.021	0.001±0.000	0.013±0.002	0.018±0.003	0.020±0.003	0.028±0.004	70.917±3.489
**P-value**	0.066	0.069	0.000	0.000	NA	0.039	0.000	0.000	NA	NA	0.000	0.017	0.285	NA	NA	NA	NA	NA	0.000

NA = not applicable.

Table 2 shows the elemental percentages among the 10 Bovidae. A few elemental percentages were significantly different across species (P<0.05): Cl was not present in Barbary sheep; Ti was only present in buffalo, greater kudu and red lechwe, and highest in buffalo; and Mn was highest in Grant’s gazelle and red lechwe. Comparing the two species of Cervidae, S, Cr, Zn and LE were higher in Sunda sambar, while aluminium (Al), P, Ca, V, Ag and Cd were highest in spotted deer (P<0.05) (Table 3). When comparing the elemental profile between antler and frontal, Al was not present in bone, while Si, Ti, Mn, Fe, Zn, Ag, Cd, Sn and Sb in antler were all significantly higher than in bone (Table 4).

**Table 2:** Mean (± SD) elemental percentages in horn of 10 species of the Bovidae family.

Species	Al	Si	P	S	Cl	K	Ca	Ti	Mn	Fe	Zn	LE
**Buffalo**	0.401±0.090	1.067±0.254	0.116±0.041	1.513±0.518	2.022±0.668	0.368±0.065	0.489±0.168	**0.011±0.001**^a^	0.005±0.000	0.066±0.066	0.014±0.001	93.637±1.009
**Barbary sheep**	0.928±0.720	3.193±1.794	0.130±0.048	1.111±0.365	0	1.029±0.549	0.744±0.188	0	0.005±0.000	0.130±0.113	0.017±0.002	91.873±1.682
**Domestic goat**	0.234±0.090	0.718±0.311	0.189±0.185	1.565±0.531	4.205±2.440	0.277±0.149	0.406±0.296	0	0.005±0.00	0.035±0.021	0.011±0.004	92.240±1.941
**Eland**	0.522±0.147	1.286±0.790	0.160±0.068	2.664±0.478	2.383±1.017	0.356±0.037	0.351±0.068	0	0.004±0.001	0.014±0.007	0.034±0.009	92.390±0.611
**Gemsbuck**	0.880±0.078	3.060±0.498	0.066±0.017	2.075±0.379	1.807±0.710	0.579±0.117	0.814±0.132	0	0.006±0.004	0.038±0.007	0.022±0.006	90.653±0.895
**Grant's gazelle**	0.397±0.056	0.896±0.521	0.108±0.028	2.005±0.561	2.140±0.122	0.403±0.112	0.687±0.153	0	**0.022±0.010**	0.017±0.007	0.034±0.011	94.544±1.657
**Greater kudu**	0.999±0.451	4.892±2.734	0.076±0.051	2.301±0.487	1.137±0.121	0.686±0.334	0.571±0.454	**0.021±0.007**^b^	0.007±0.002	0.051±0.025	0.022±0.001	89.993±3.797
**Nyala**	1.930±0.123	4.205±0.292	0.098±0.013	2.434±0.491	2.184±0.329	0.389±0.060	0.482±0.047	0	0.005±0.001	0.061±0.018	0.026±0.001	89.530±0.598
**Red lechwe**	0.555±0.048	2.174±0.251	0.107±0.009	2.543±0.528	2.730±1.288	0.505±0.018	0.491±0.108	**0.019±0.001**^b^	**0.025±0.004**	0.030±0.013	0.021±0.005	90.840±1.156
**Sitatunga**	1.753±0.704	4.093±2.142	0.075±0.035	2.792±1.434	1.286±0.164	0.464±0.438	0.240±0.094	0	0.005±0.000	0.135±0.122	0.020±0.003	89.960±1.345

Bold indicates a significant differences at P-value<0.05 when compared among the same element.

^a,b^ represent the catagorised group based on P-value<0.05.

**Table 3:** Mean (± SD) element percentages in antlers of two Cervidae species.

Species	Al	Si	P	S	K	Ca	Ti	V	Cr	Mn	Fe	Zn	Zr	Ag	Cd	Sn	Sb	LE
**Sunda sambar**	0.638±0.236	2.439±1.030	5.507±1.206	0.335±0.100	0.341±0.136	16.266±1.451	0.048±0.006	0.013±0.003	0.009±0.001	0.026±0.009	0.082±0.011	0.037±0.024	0.001±0.001	0.012±0.002	0.016±0.002	0.019±0.003	0.027±0.005	73.966±2.404
**Spotted deer**	1.033±0.202	2.420±1.431	7.438±0.702	0.160±0.031	0.916±0.506	20.313±0.902	0.060±0.012	0.020±0.005	0	0.030±0.010	0.082±0.064	0.013±0.001	0.001±0.001	0.015±0.001	0.021±0.001	0.021±0.002	0.027±0.005	68.660±1.963
**P-value**	0.015	0.492	0.011	0.007	0.052	0.001	0.079	0.036	NA	0.243	0.497	0.045	0.363	0.006	0.002	0.211	0.494	0.004

NA = not applicable.

**Table 4:** Mean (± SD) element percentage between antler and frontal bone in samples combined across Cervidae species.

Specimen	Al	Si	P	S	K	Ca	Ti	V	Cr	Mn	Fe	Zn	Ag	Cd	Sn	Sb	LE
**Antler**	0.813±0.294	2.432±1.092	6.365±1.396	0.257±0.118	0.597±0.444	18.064±2.430	0.053±0.010	0.016±0.005	0.005±0.005	0.028±0.009	0.082±0.040	0.027±0.021	0.013±0.002	0.018±0.003	0.020±0.003	0.027±0.004	71.608±3.49
**Frontal Bone**	0	0.215±0.149	5.954±0.553	0.288±0.099	0.318±0.246	17.331±0.846	0.021±0.013	0.015±0.006	0.003±0.004	0.006±0.004	0.029±0.015	0.005±0.003	0.016±0.002	0.021±0.002	0.023±0.002	0.032±0.003	75.402±1.36
**P-value**	NA	0.000	0.215	0.279	0.062	0.206	0.000	0.283	0.178	0.000	0.009	0.000	0.009	0.014	0.009	0.014	0.006

NA = not applicable

**Fig 1:**
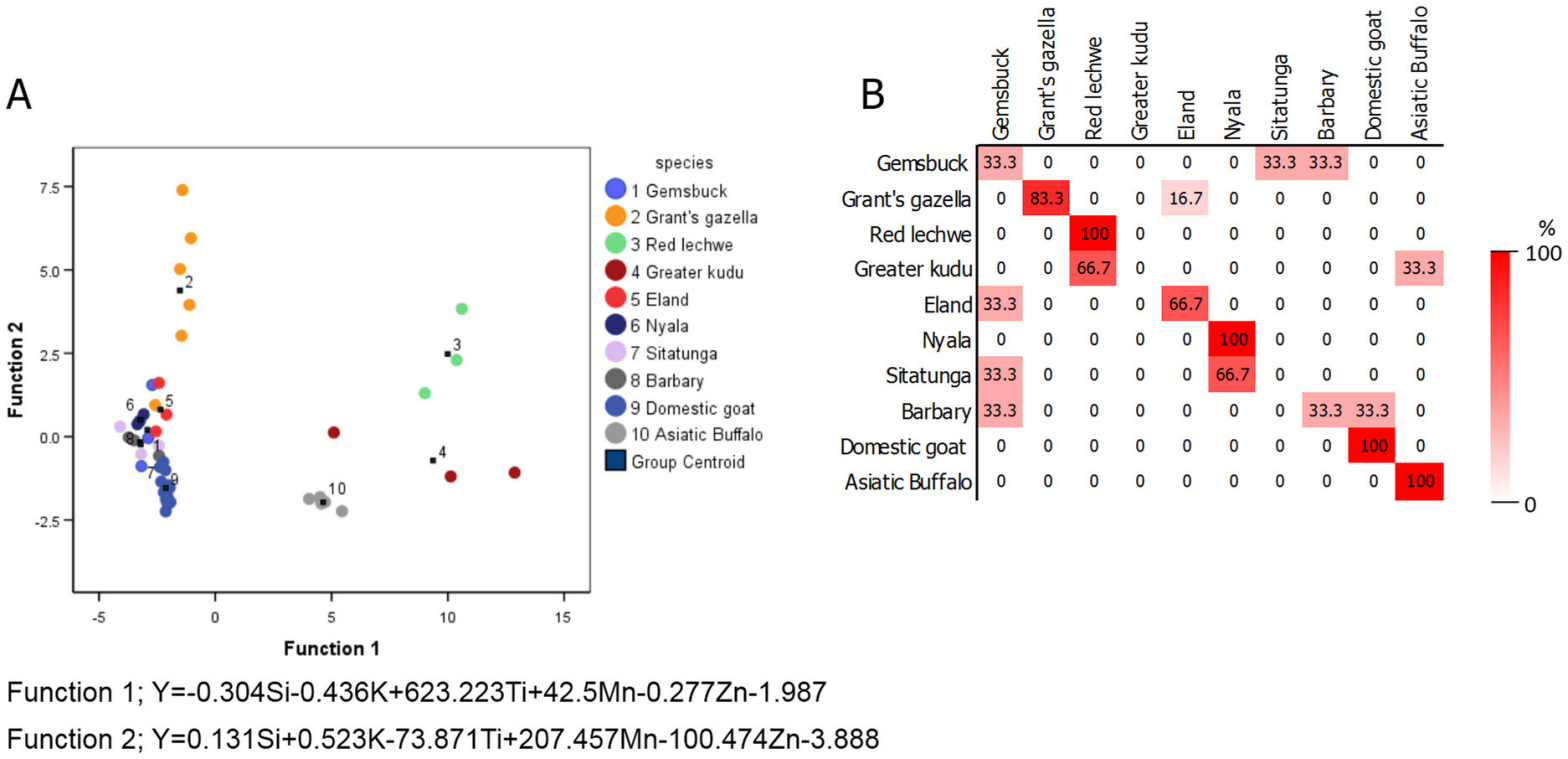
The feasibility of elemental composition in horn for species classification. (A) Canconical discriminant function plots of the elemental composition of horn of different species (B) the classification results by discriminant analysis expressed as a percentage of the correct prediction.

The elemental data obtained from Bovidae and Cervidae were analyzed by stepwise discriminant analysis for differentiating species by horn or antler. The equation for species predication of horn is provided in Fig 1; Eq 1 was determined to predict species by antler.

 Y=1196.31Cr−6.92 (1)

Elemental profiling by XRF analysis distinguished between antler species (i.e. Sunda sambar and spotted deer) with 100% accuracy (Table 5). Distinction among Bovidae species proved 75% accurate, with 100% accuracy in discriminating among domestic goat, red lechwe, nyala and Asiatic buffalo (Fig 1A and 1B).

**Table 5:** Classification result of species prediction using elemental profiling of antlers from two species of Cervidae.

	Sunda sambar	Spotted deer
Sunda sambar	7 (100)	0
Spotted deer	0	4 (100)

### Study 2: Elemental analysis of teeth from multiple species

The elemental percentages in teeth varied among eight different species (Table 6). Ten elements were found in all species: Si, P, Ca, Ti, Mn, Fe, Ag, Cd, Sn and Sb. Elephant, human and monkey were the only species to contain Al in teeth. The highest percentages of K were found in elephant and horse (P<0.05). Copper (Cu) was significantly higher in dog compared to all other species. Ni was found in dog, human and monkey, and significantly higher in dog. Zn was significantly higher in monkey and dolphin compared to all other species. Mn was highest in deer compared to all other species (P<0.05). Some elements were not detected at all in certain species: Zr in monkey, Cr in dolphin, V and Cu in dolphin and crocodile, Zn in crocodile, and S in deer and dog.

**Table 6:** Mean (± SD) percentage of elements in teeth of eight species.

Species	Al	Si	P	S	K	Ca	Ti	V	Cr	Mn	Fe	Ni	Cu	Zn	Zr	Ag	Cd	Sn	Sb	LE
**Deer**	0	0.310±0.058	12.310±1.524	0	0	28.307±2.305	0.054±0.005	0.026±0.010	0.009±0.005	**0.173±0.105**	0.393±0.102	0	0.002±0.002	0.013±0.003	0.002±0.001	0.021±0.003	0.027±0.003	0.031±0.001	0.040±0.003	58.277±3.831
**Dog**	0	0.310±0.131	12.095±2.000	0	0	26.298±2.112	0.045±0.014	0.009±0.010	0.004±0.007	0.038±0.021	0.217±0.112	**0.023±0.300**	**0.053±0.031**	0.028±0.022	0.001±0.000	0.019±0.003	0.025±0.004	0.027±0.006	0.033±0.007	60.712±3.883
**Elephant**	0.337±0.120	1.010±0.617	6.635±2.791	0.441±0.240	**0.981±0.710**	20.933±4.540	0.043±0.012	0.018±0.009	0.009±0.007	0.010±0.006	0.041±0.020	0	0.005±0.003	0.012±0.006	0.002±0.001	0.016±0.003	0.022±0.004	0.023±0.004	0.031±0.006	69.857±7.147
**Horse**	0	0.946±0.304	11.143±1.757	**0.952±0.200**	0.302±0.025	28.803±3.981	0.052±0.016	0.014±0.004	0.020±0.001	0.023±0.004	0.051±0.013	0	0.003±0.000	0.018±0.004	0.004±0.001	0.017±0.003	0.023±0.004	0.028±0.004	0.034±0.005	57.687±5.576
**Human**	0.146±0.150	0.469±0.400	12.900±1.213	0.071±0.072	0	29.824±2.083	0.050±0.008	0.020±0.009	0.105±0.300	0.028±0.026	0.076±0.040	0.001±0.001	0.002±0.001	0.027±0.020	0.001±0.001	0.020±0.002	0.027±0.002	0.030±0.003	0.040±0.004	56.067±3.079
**Monkey**	0.280±0.241	0.508±0.399	14.218±0.679	0.090±0.080	0	29.933±1.708	0.060±0.008	0.025±0.003	0.014±0.003	0.068±0.035	0.100±0.055	0.001±0.001	0.004±0.003	**0.067±0.016**^a^	0	0.024±0.001	0.032±0.001	0.037±0.002	**0.049±0.002**^a^	54.519±2.540
**Dolphin**	0	0.268±0.048	11.553±0.571	0.113±0.013	0	22.120±0.481	0.017±0.010	0	0	0.008±0.001	0.036±0.012	0	0	**0.067±0.009**^a^	0.002±0.000	0.013±0.001	**0.014±0.002**	**0.014±0.002**	**0.016±0.001**^a^	65.847±0.931
**Crocodile**	0	0.664±0.146	9.700±1.825	0.095±0.014	0	23.510±2.862	0.038±0.001	0	0.005±0.009	0.015±0.002	0.237±0.356	0	0	0	0.002±0.000	0.019±0.002	0.026±0.002	0.029±0.002	0.035±0.001	65.640±4.965

Bold indicates a significant differences at P-value<0.05 when compared among the same element.

^**a**^Indicates values that are not different when compared to bold.

**Fig 2:**
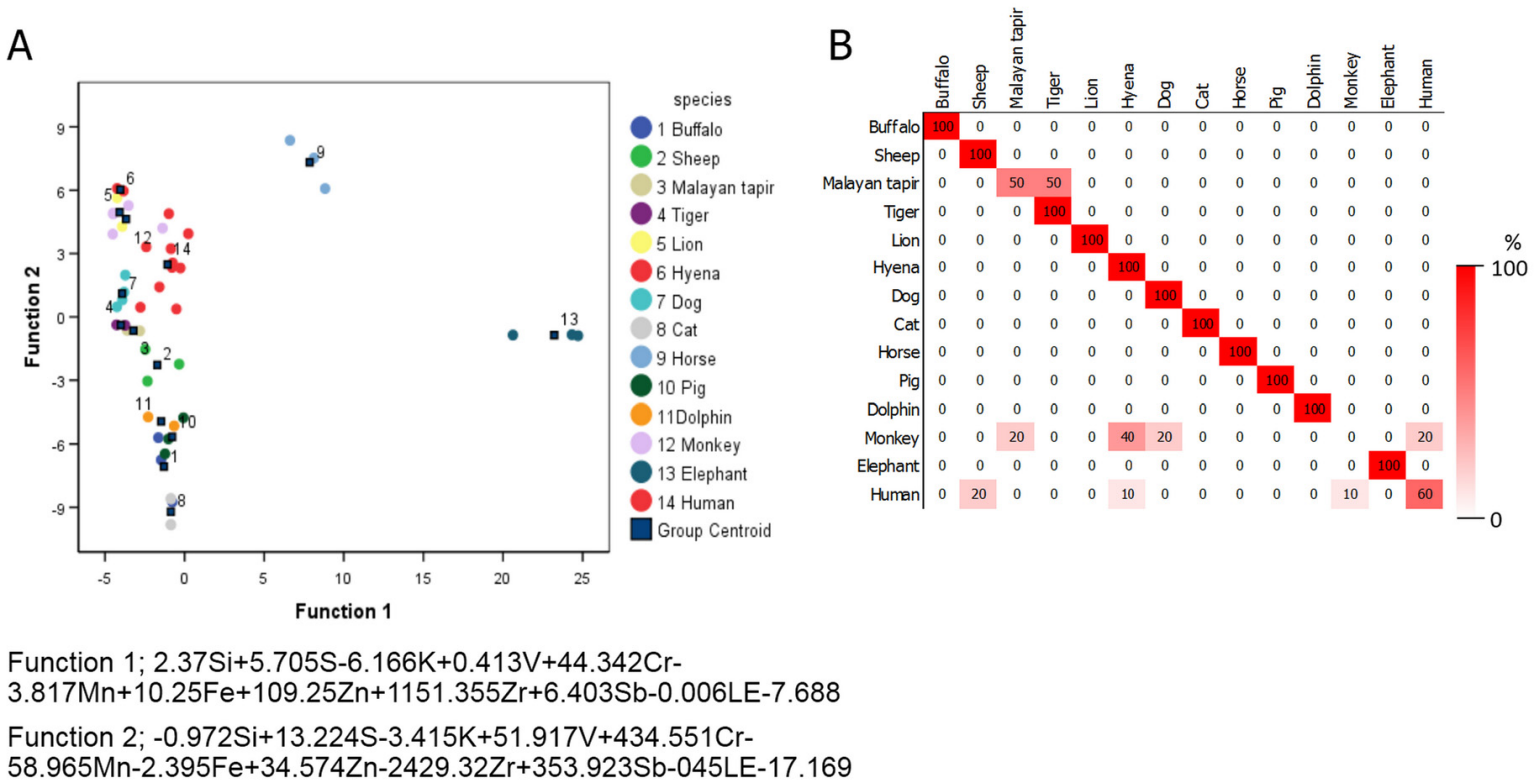
The feasibility of elemental composition in molar teeth for species classification. (A) Canconical discriminant function plots of the elemental composition of molar teeth of different species and (B) the classification results by discriminant analysis expressed as a percentage of the correct prediction.

**Fig 3:**
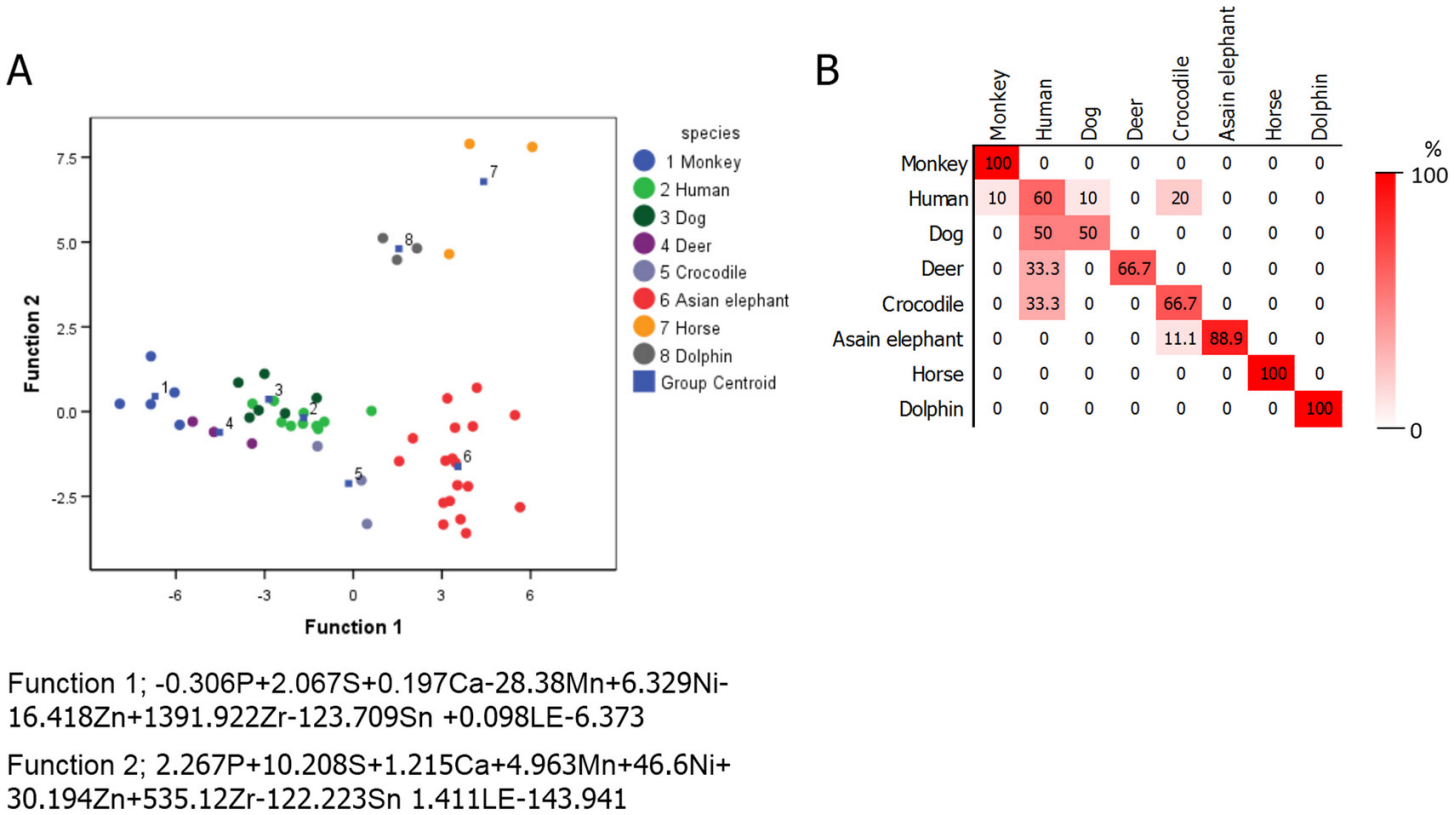
Differentiation of human and others from elemental composition in mineralized tissues. Canconical discriminant plots of the elemental composition of human, monkey and non-primate (dog, Asian elephant, horse and spinner dolphins) teeth (A) and humerus bone (B).

Canconical discriminant plots derived from molar teeth across six species found 78.4% correct discrimination, with monkey, horse and dolphin showing complete discrimination from other species (Fig 2A and 2B). Comparing human specifically to the other species (primate and non-primate bones), a correct prediction of 75% was obtained. Humerus bones of human were however misidentified and predicted as monkey (Fig 3A). Misidentification is likely attributed to human largely overlapping with other non-primates, and to a lesser extent with monkey.

### Study 3: Elemental analysis of humerus bone from 14 species

Elemental composition in humerus bone varied among 14 species (Table 7). Ten elements were presented in humerus of all species: P, Ca, Mn, Fe, Zn, Ag, Cd, Sn, Sb and LE. Elephant, horse, human, monkey and pig contained Al, with elephant having significantly higher percentages. Si and Ti were not present in dolphin; Si in elephant and pig were significantly higher compared to other species. Pig and dolphin showed significantly lower percentages of P and Ca compared to all other species. S was the highest in horse and dolphin (P<0.05). V was highest in buffalo, but was not detected in cat, dog, horse and pig. Four species did not contain Cr in humerus: buffalo, cat, dog and sheep. Mn and Fe were significantly higher in elephant, and Zn was significantly higher in horse and dolphin. Zr was not presented in dog, hyena or monkey. Sn was found to be significantly higher in elephant, while Sb was lowest in cat. Light elements were significantly higher in pig and dolphin.

**Table 7:** Mean (± SD) percentage of elements humerus bone of 14 species.

Species	Al	Si	P	S	K	Ca	Ti	V	Cr	Mn	Fe	Zn	Zr	Ag	Cd	Sn	Sb	LE
**Buffalo**	0	0.397±0.083	6.071±0.297^a^	0.168±0.050	0	22.233±0.364	**0.045±0.009**^a^	**0.027±0.004**	0	0.012±0.002	0.011±0.004	0.011±0.001	0.003±0.000	0.017±0.000	0.021±0.001	0.024±0.000	0.030±0.001	**70.677±0.538**
**Cat**	0	0.526±0.011	11.448±0.160	0.034±0.003	0	23.685±0.501	**0.028±0.002**^a^	0	0	0.008±0.001	0.008±0.001	0.024±0.001	0.003±0.000	0.013±0.001	0.017±0.000	0.018±0.001	**0.022±0.002**	64.405±0.839
**Dog**	0	0.096±0.021	10.753±0.255	0	0	24.698±0.661	**0.033±0.006**^a^	0	0	0.059±0.001^a^	0.242±0.005	0.013±0.001	0	0.016±0.001	0.020±0.000	0.022±0.001	0.027±0.001	63.830±1.051
**Elephant**	**0.416±0.016**	**1.460±0.306**^a^	8.523±0.751	0.191±0.023	0.000	22.577±0.892	**0.059±0.005**^a^	0.023±0.002^a^	0.010±0.002	**0.068±0.023**	**1.926±0.361**	0.024±0.011	0.003±0.000	0.019±0.000	0.025±0.001	**0.028±0.000**	0.036±0.002	64.607±1.645
**Horse**	0.170±0.010^a^	0.466±0.127	10.403±0.215	**0.394±0.072**^a^	0	24.727±0.146	**0.034±0.004**^a^	0	0.013±0.002	0.026±0.007	0.193±0.040	**0.065±0.008**^a^	0.002±0.000	0.017±0.002	0.025±0.005	0.026±0.002^a^	0.031±0.001	63.823±0.545
**Human**	0.069±0.100	0.440±0.446	9.159±1.180	0.227±0.073	0.094±0.178	23.104±1.084	**0.031±0.005**^a^	0.014±0.005	0.004±0.002	0.013±0.006	0.112±0.061	0.028±0.007	0.001±0.000	0.016±0.001	0.021±0.001	0.023±0.002	0.030±0.002	66.530±1.715
**Hyena**	0	0.144±0.075	10.460±0.240	0.083±0.000	0	24.275±0.205	**0.031±0.007**^a^	0.015±0.002^a^	0.011±0.000	0.015±0.000	0.077±0.009	0.016±0.005	0	0.016±0.001	0.021±0.002	0.023±0.000	0.029±0.001	64.795±0.544
**Lion**	0	0.108±0.031	10.640±0.226	0.070±0.017	0	23.900±0.255	**0.030±0.000**^a^	0.019±0.001^a^	0.011±0.001	0.035±0.003^a^	0.074±0.000	0.010±0.000	0.001±0.000	0.016±0.000	0.019±0.002	0.024±0.000	0.030±0.002	65.020±0.438
**Malayan tapir**	0	0.540±0.046^a^	8.174±1.295	0.194±0.001^a^	0.442±0.042	23.270±0.297	**0.037±0.09**^a^	0.008±0.011	0.009±0.000	0.015±0.001	0.019±0.003	0.019±0.002	0.002±0.000	0.016±0.000	0.021±0.001	0.024±0.001	0.031±0.000	67.395±1.223^a^
**Monkey**	0.229±0.314^a^	0.534±0.457	9.268±0.661	0.265±0.150^a^	0.210±0.426	23.118±1.312	**0.030±0.005**^a^	0.008±0.005	0.007±0.005	0.032±0.029	0.080±0.053	0.015±0.009	0	0.014±0.001	0.019±0.001	0.020±0.002	0.026±0.002^a^	66.123±1.730
**Pig**	0.220±0.010^a^	**1.356±0.103**^a^	**3.917±0.216**^a^	0.153±0.008	0.238±0.096	**16.083±0.680**^a^	**0.040±0.002**^a^	0	0.010±0.002	0.009±0.000	0.027±0.021	0.021±0.002	0.001±0.000	0.014±0.002	0.020±0.003	0.021±0.002	0.029±0.002	**78.287±0.735**^a^
**Sheep**	0	0.171±0.006	7.097±0.660	0.072±0.014	0	23.793±1.516	**0.033±0.024**^a^	0.022±0.006^a^	0	0.008±0.001	0.070±0.091	0.028±0.004	0.001±0.000	0.017±0.000	0.022±0.002	0.025±0.001^a^	0.032±0.001	68.443±2.168^a^
**Tiger**	0	0.118±0.007	11.105±0.700	0.082±0.001	0.341±0.018	24.535±1.336	**0.037±0.011**^a^	0.000	0.011±0.000	0.011±0.001	0.045±0.017	0.023±0.005	0.002±0.000	0.015±0.001	0.020±0.002	0.021±0.002	0.026±0.001	63.800±1.782
**Dolphin**	0	0	**4.278±0.840**^a^	**0.445±0.065**^a^	0.450±0.139	**15.160±1.245**^a^	0	0.021±0.001^a^	0.000	0.011±0.001	0.019±0.004	**0.047±0.002**^a^	0.001±0.000	0.014±0.003	0.019±0.000	0.018±0.001	0.026±0.001^a^	**79.470±2.121**

Bold indicates a significant differences at P-value<0.05 when compared among the same element.

^**a**^Indicates values that are not different when compared to bold.

**Fig 4:**
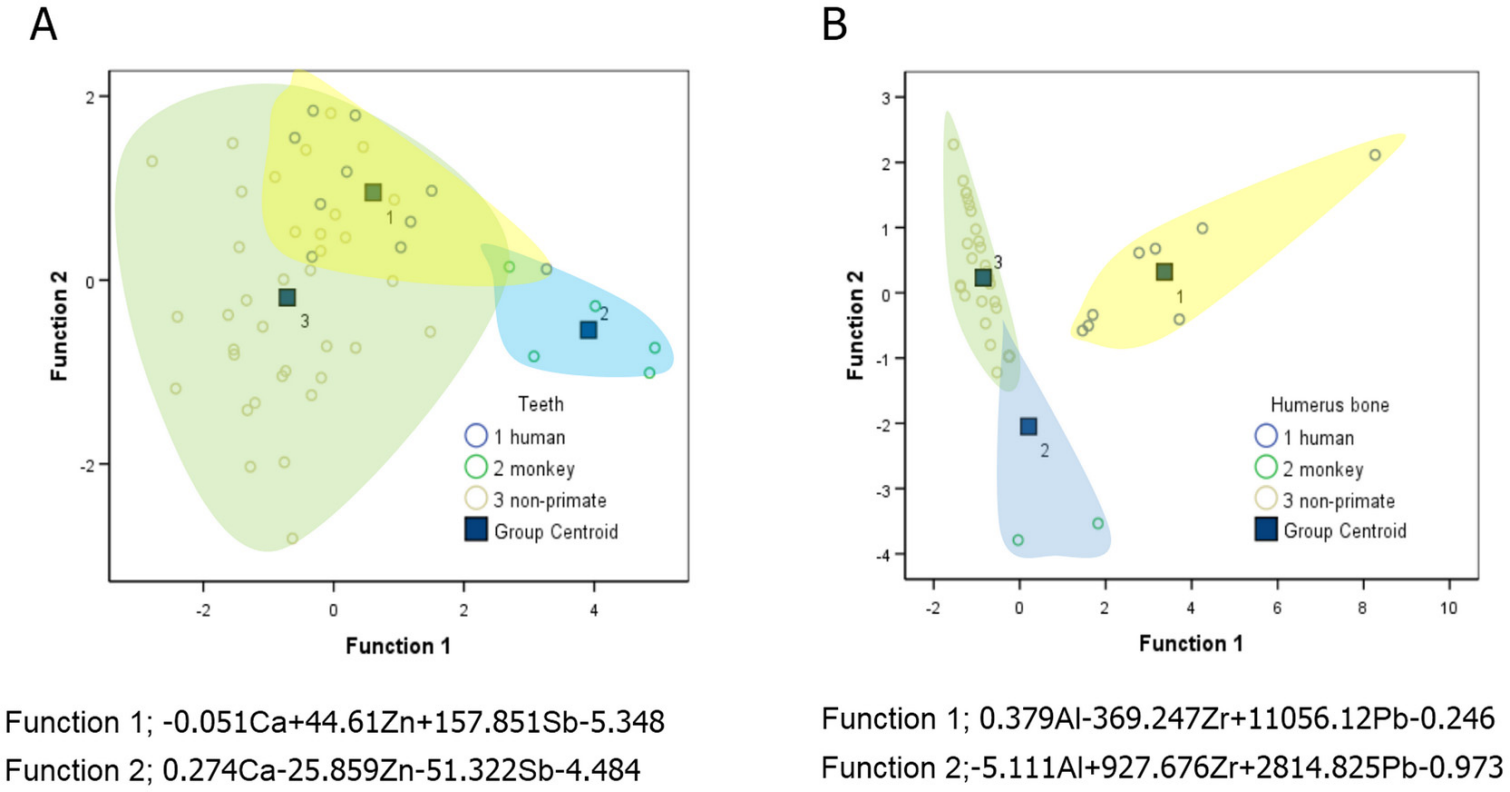
The feasibility of elemental composition in humerus bone for species classification. Canconical discriminant function plots of the elemental composition of humerus bone of different species (A) and the classification results by discriminant analysis expressed as a percentage of the correct prediction (B).

Results of stepwise discriminant analysis is shown in Fig 4A. Overall, the correct prediction rate for species identification was 79.2% (Fig 4B). All species analyzed except Malayan tapir, monkey and human, exhibited a 100% correct species identification by cross-validation method (Fig 4B). Furthermore, element content in teeth was used to distinguish between human and other species, with the elemental composition of teeth correctly discriminating human teeth from other species with 80% accuracy. However, 22.2% of the time non-primate teeth were misclassified as human based on elemental composition (Fig 3B).

**Fig 5:**
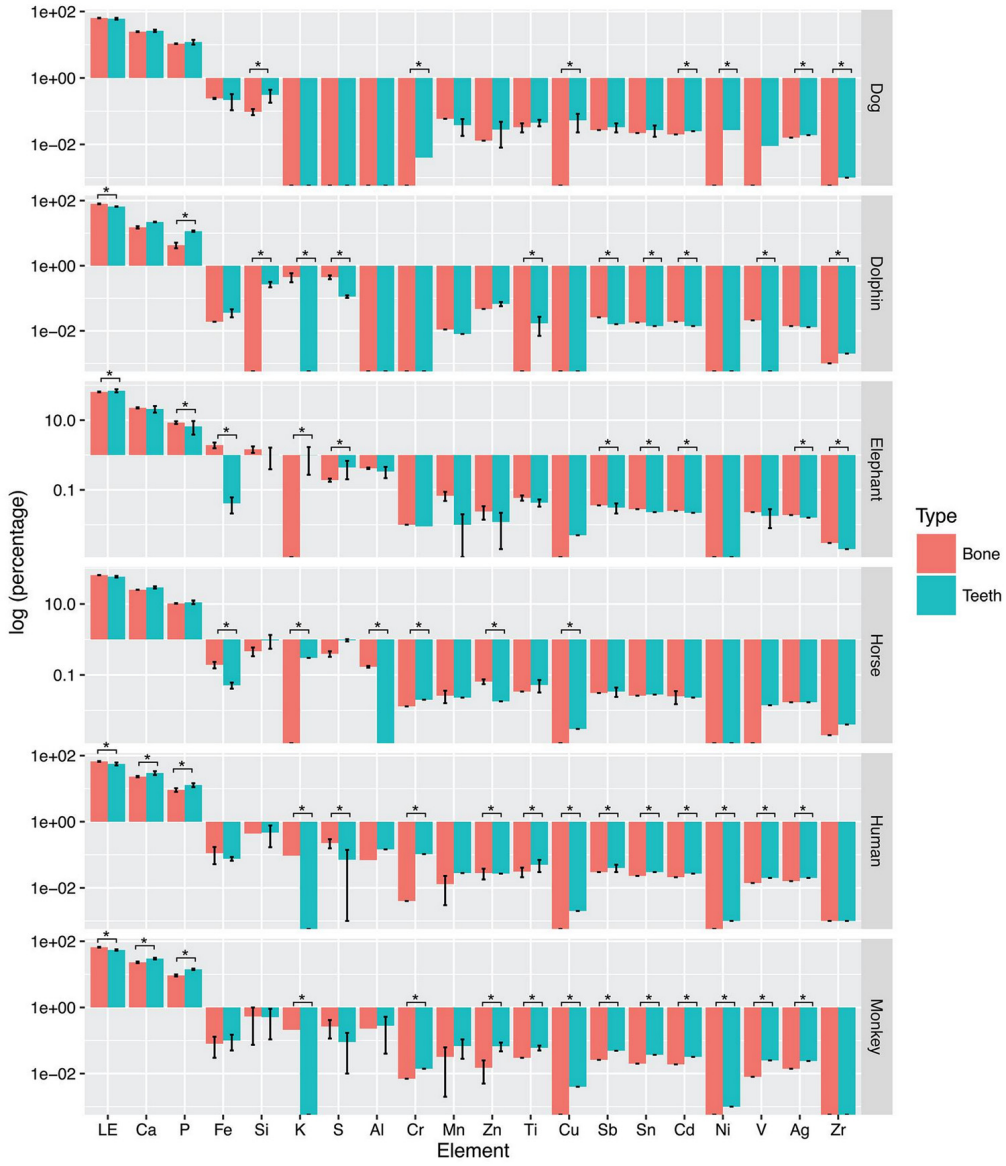
The percentage of multiple elements in various species. The bar express mean (± standard deviation (SD)) percentages of elements in dog, elephant, horse, human, monkey and dolphin teeth and bone. * indicates a significant difference at P<0.05.

Intra-species comparison of teeth and humerus bone demonstrated both monkey and human had the most elemental variability between the two mineralized tissues, with 14 out of the 20 elements differing between teeth and humerus (Fig 5). In contrast, the horse showed the lease elemental variability, with 5 out of the 20 elements overlapping between teeth and humerus. Dog had eight elements (Si, V, Cr, Ni, Cu, Zr, Ag and Cd), elephant had 11 elements (P, S, K, Fe, Zn, Zr, Ag, Cd, Sn, Sb and LE), horse had five elements (Al, K, Cr, Cu and Zn), human had 14 elements (P, S, K, Ca, Ti, V, Cr, Ni, Cu, Ag, Cd, Sn, Sb and LE), monkey had 14 elements (P, K, Ca, Ti, V, Cr, Ni, Cu, Zn, Ag, Cd, Sn, Sb and LE), and dolphin had 11 elements (P, Si, S, K, Ti, V, Zr, Cd, Sn, Sb and LE) that differed significantly between teeth and bone.

**Fig 6:**
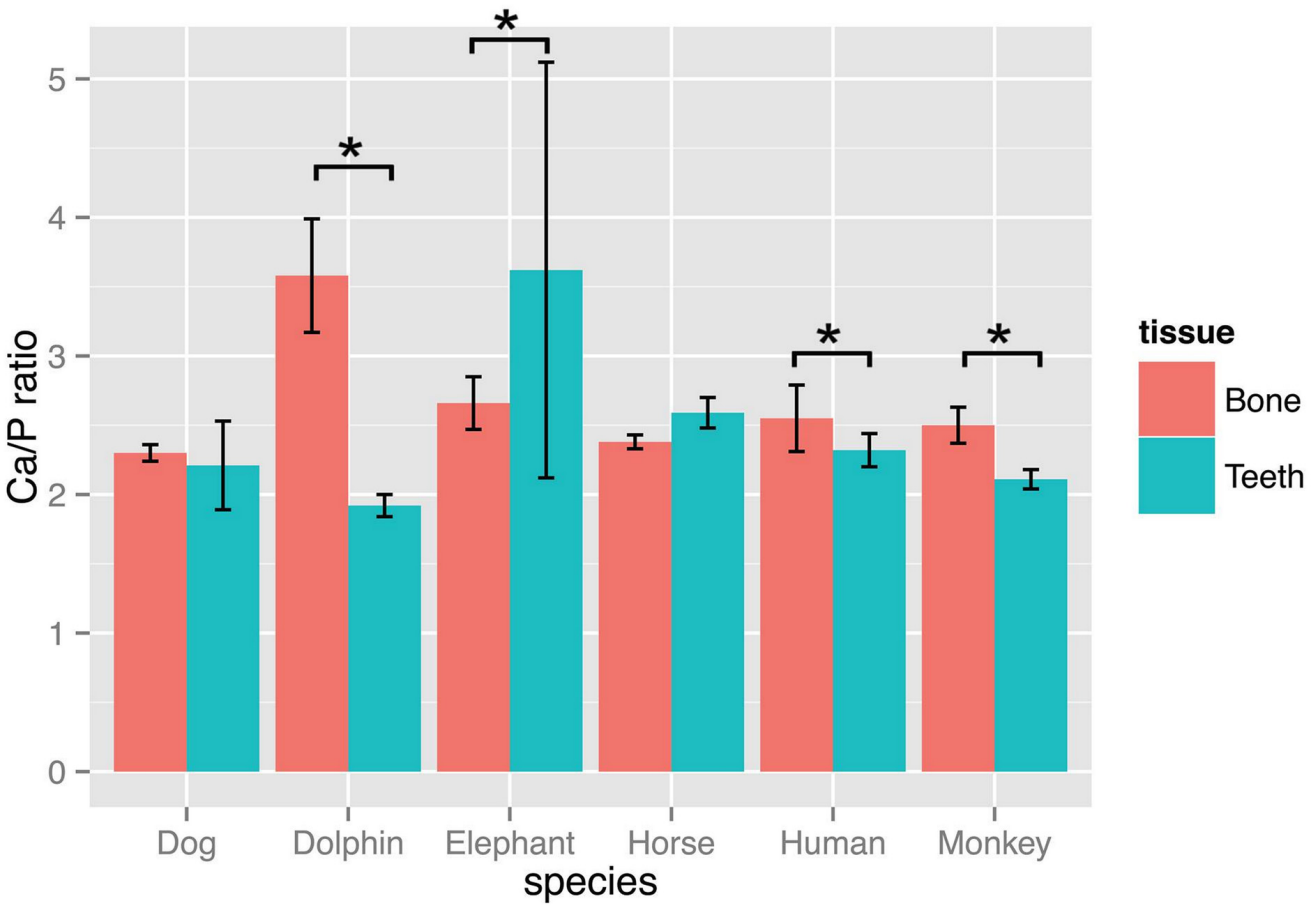
The ratio of Ca to P between teeth and bone across various species. The bar express mean (± SD) of Ca/P ratios between teeth and bone of dolphin, human, horse, dog, monkey and elephant. * indicates a significant difference at P<0.05 within the same species.

The ratio of Ca/P was calculated and presented in Fig 6. The average of Ca/P of humerus and teeth among mammal species was 2.66 and 2.46, respectively. Four species (dolphin, human, monkey and elephant) showed a statistically significant difference between teeth and humerus. In dolphin, human and monkey, the Ca/P in humerus bone was greater than that in teeth; however, this relationship was reversed in elephant.

## Discussion

This was the first comparative study to explore the accumulation of multiple elements in mineralized tissues: horns, antlers, humerus and teeth of various animal species and found significant differences that may be related to structure and function, and potentially aid in forensic classification of species. In addition to their importance in body scaffolding, food digestion, mate attraction, and defense, these tissues also act as mineral storage sites to support biological activities involving enzymes, signaling molecules and homeostasis. Of particular interest was the remarkable difference in elemental distribution between horn and antler, even within more closely related species. The hunting of animals for these products is leading to population declines for many species. Moreover, we found that many elements in the humerus bone varied significantly across species; for example, Fe accounted for the highest proportion in the Asian elephant humerus as compared to 14 other species. Collectively these results suggest that the elemental content of mineralized tissues may be useful for species differentiation and/or can be used for forensic classification and biological conservation.

### Differences between horn and antler

There are several types of cranial appendages found in mammals, including antlers (in cervids), horns (in bovids), pronghorns (in antelope) and ossicones (in giraffids) [21]. Each may differ in elemental qualities due to their developmental origins. Horns are composed of an outer keratin layer, covering the core of live bone that grows out of the frontal bone of the skull. The dermis is continuous with the periosteum and epidermis, which keratinizes and forms the protective covering of the horn, not unlike that of a claw, skin or hair [22]. Keratin is composed of either a fibrous, keratin filament or an amorphous, intermediate filament protein formed by epidermal cells differentiating into cornified or keratinized cells, respectively [23]. Many components are required for keratinization to occur properly [22], including amino acids (cysteine, histidine and methionine), minerals (Ca, Zn, Cu, selenium (Se) and Mn) and vitamins A, D, E and biotin.

Our study identified Al, Si, P, S, Cl, K, Ca, Ti, Mn, Fe and Zn in the horns of 10 species, with the major constituents being Cl, S and Si. Zhang and colleagues [24] evaluated the elemental content in three Bovidae: buffalo, cattle and sheep using inductively coupled plasma-atomic emission spectroscopy (ICP-AES). Eighteen elements, including Al, B, Ba, Ca, Cd, Co, Cr, Cu, Fe, Mg, Mn, (molybdenum) Mo, Ni, P, (strontium) Sr, Ti and Zn, were detected, with Ca, P and Zn being the major components. The difference in proportion of elements between the two studies may be due to the different sampling techniques. ICP-AES scans the elemental distribution of the entire depth of the horn, whereas handheld XRF detects only the elemental composition of the horn’s surface. Speculatively, the elemental composition may be not be homogenous in cross-sectional areas of the horn, leading to varied elemental content between layers of keratinized tissue. Indeed, our previous work has shown that elemental distributions can vary across longitudinal and transversal sites of various tooth and bone types [3,12,13]. Finally, the difference in results may be related to the specific elements measured in each study. Keratinization requires key elements (e.g. Zn, Cu, Se, Mn) for normal formation to ensue [22]; however, in the present study, neither Cu nor Se were measured in the horn, altering the overall percentage of total elements detected.

Elemental composition was predominantly the same in the 10 Bovidae species analyzed in this study. Of the elements detected, only Ti and Mn varied across species. Ti was found only in buffalo, greater kudu and the red lechwe, with lowest levels detected in the buffalo. The literature is still unclear regarding the physiological effect of Ti in the body [1]. Although Mn was found in all 10 species, Mn was significantly higher in Grant’s gazelle and red lechwe compared to all other species. Similar to Ti, the purpose of Mn in the horn is unknown. Si and Cl were present at the highest proportions out of the 11 elements (not including LE). Cl was the major element in buffalo, goat, eland, Grant’s gazelle and red lechwe, whereas Si was the major element in Barbary sheep, gemsbok, greater kudu, nyala and sitatunga. Although the physiological function of Cl in horn is unknown. Si is known to be involved in collagen synthesis [1]. However, the molecular composition of horn lacks collagen. The detected Si in horn is speculatively due to environmental contamination, as soil and water are rich in Si [25]. As such, the different amounts of detected Si across species may reflect the animals’ geographical distribution.

In contrast to horn, antlers have a microstructure and chemical composition similar to bone, primarily being comprised of type I collagen and minerals [26]. As a result, a number of elements differed between antler and horn. In fact, of the 19 elements detected, only Al, Si and Zn were similar between the two. A number of elements were observed only in horn (Cl) or antler (V, Cr, Zr, Ag, Cd, Sn, Sb). The detection of heavy metals in the antler may be due to the diet of Cervidae species, which ingest soil to meet their salt and mineral requirements. As a consequence, heavy metals in the soil are also ingested. The major differences observed between the antler and horn were related to the Ca and P composition, such that Ca and P were observed to be 30- to 40-fold higher in the antler compared to the horn. These results are expected as antler contains a high proportion of hydroxyapatite similar to bone, compared to the keratinized tissue composition of the horn’s surface.

### Elements in teeth and bone

Teeth and bone are both mineralized tissues that consist mainly of hydroxyapatite [27]. By handheld XRF, we detected 20 and 17 elements in teeth and humerus bone, respectively, of different animal species. Only eight elements (S, K, Mn, Ni, Cu, Cd, Sn and Sb) differed across species. For example, the proportion of Ni and Cu was highest in dog, but lower in other species. K was found in elephant and horse, but was undetectable in the other species. S comprised the greatest proportion of elements in horse, whereas in deer it was Mn. In another comparative study, de Dios Teruel [28] found eight elements (Si, P, S, K, Ca, Fe, Cu and Zn) differed between human, bovine, porcine and ovine teeth, some of which were similar to our study results. A difference in the elemental content between deciduous and permanent teeth from elephants was noted [3], which is in accordance with the accumulation of elements, especially metal elements (Pb, Cd, Cu and Cr), in deciduous teeth decreasing with age in children [29, 30]. The concentrations of elements in teeth can thus be used to estimate age and infer the type of tooth. Additionally, elemental concentration in teeth can distinguish between an Asian or African elephant tusk [31]. Not surprisingly, Ca and P were the most abundant elements in teeth of all species, a finding well documented in other species [29, 32].

In humerus bone, all but five elements (K, Cr, Zr, Ag and Cd) differed significantly among species. Most of the elements in these bones were due to accumulation in the structure of hydroxyapatite. Numerous substitution metals can be found in hydroxyapatite crystals; metal cations such as K^+^, Na^+^, Mn^2+^, Ni^2+^, Cu^2+^, Mg^2+^ or Zn^2+^ can replace Ca^2+^, whereas anionic complexes, including AsO_4_^3-^, SO_4_^2-^, CO_3_^2-^ or SiO_4_^4-^ can provide a substitute for PO_4_^3-^, and anions such as Cl^-^ or F^-^can occupy OH^-^ in the crystal structure [33–35]. Therefore, it is not surprising these elements were found in our studied samples: antlers, humerus and teeth. A few elements demonstrated specific-species accumulation: Al, Si, S, K, V, Cr, Zr, indicating different species often have an elemental accumulation bias, which could be due in part to inherent species differences. *In vitro* studies using synthetic hydroxyapatite and bovine bone meal, provided evidence for the immobilization or incorporation of Pb^2+^, Zn^2+^, Sr^2+^ and other divalent metal ions into hydroxyapatite [34, 36, 37] via four pathways: 1) ion exchange process; 2) surface complexation; 3) dissolution and precipitation; and 4) co-precipitation [36]. These pathways can be expected to be similar for other divalent ions. Moreover, when compared to other tissues, the main source of bodily accumulation of Zn and Pb is the femur bone [38]. An interesting finding was Fe in elephants, where Fe proportion was higher by an order of magnitude in bone (humerus), but not in teeth, when compared to other species. The long bone of the elephant is different from other species because it has no bone marrow cavity. In other species, bone marrow contains hemopoietic cells for manufacturing blood cells and is composed of a network of dense cancellous bone with hemopoietic cells [39]. The lack of a bone marrow cavity may be the reason for the increased proportion of Fe found in the humerus bones of elephants. Other species differences may have a physiological basis; for example, the lack of Al, Ti and Cr in dolphin may be because these elements are less than 0.001 ppm at 3.5% salinity in seawater [40, 41].

Apart from species contributing to variation in elemental content, diet and environmental also can affect bodily element composition [42–44]. Primary teeth of Ugandan compared to British children vary in elemental concentrations [42]. The authors concluded the primary reason for difference in elemental concentrations was environmental factors; for example, the Ugandan diet consisted primarily of cassava tubers, with elemental deficiencies resulting in malnutrition. Specific elements can even be used to track animals: strontium (Sr) concentrations in teeth were used successfully to track deer to specific regions based on the relative abundance of the Sr isotope in different regions [44].

To examine the elemental content between teeth and bone among six animal species (dog, horse, monkey, elephant, human and dolphin), we evaluated the percentage of the two major elements as a Ca/P ratio. One application of the Ca/P ratio is as an indication of bone or teeth strength [1, 45]. Within species, the Ca/P ratio was highest in bone compared to teeth for the two primate species (monkey and human), with the opposite observed in the elephant. Asian elephants are both grazers and browsers, and in addition to grass also will consume tree bark, branches, and soil [46, 47]. Further, the anatomy of elephant teeth differs in several ways from that of other species [48]. Elephant teeth are polyphyodonts that have cycles of tooth rotation throughout their lives. The chewing teeth are replaced six times in a lifetime. Teeth are not replaced by new ones emerging from the bone vertically as in most mammals, but rather new teeth grow in at the back of the mouth and move forward to push out the old ones. Collectively, this may partially explain why teeth are stronger than bone in elephants. Interestingly, dolphin had the highest Ca/P ratio. This finding cannot be fully explained. However, we speculate it may be related to the aquatic environment and the dolphin’s locomotion, mimicking continuous exercise, which would promote greater bone turn over and bone matrix accumulation.

### Species discrimination using elemental content

The large dataset of elemental profiles of horn, antler, teeth and humerus bone allowed for discriminant analysis, which was used to determine XRF’s ability to distinguish among samples of different animal species. In antler, it was possible to discriminate between Sunda sambar and spotted deer with 100% accuracy due to Cr being present only in sambar deer. The discrimination of horn, teeth and humerus among all species was 75%, 78.4% and 79.2%, respectively. Using elements in horn for species differentiation had the lowest success rate primarily because the external surface of horn is not true bone, leading to a low and highly variable accumulation of elements. Thus, additional methods may be required in identifying horn species. Recently, Zhang [24], demonstrated that the horn of three domestic bovines (buffalo, cattle and sheep) could be distinguished based on the conformation of keratin and its related peak on the Raman spectra. Moreover, the structure of keratin is not influenced by the animal’s food intake, leading to less variation compared to other mineralized tissues. The elemental profile of the humerus bone proved more accurate compared to teeth for species discrimination. Among the humerus bone data of 14 species, except for Malayan tapir, monkey and human, all other humerus bone samples showed a 100% accuracy in predicting the species of origin correctly. Taken together, the elemental profile of humerus bone was the most reliable for distinguishing among species. We also established a discriminant function for distinguishing between human and non-human based on teeth or humerus bone. The humerus bone showed a greater ability to discriminate human from non-human samples compared to teeth. It has previously been shown that compared to non-human bone, human compact bone can by identified by the osteon structure [49, 50]. Geometric morphometry also has been used to effectively quantify complex skeletal differences, such as those found among hominid temporal bones [51]. However, the disadvantage of using either the osteon structure or geometric morphometry is that it may not be as effective with broken bones or incomplete samples. Conversely, the elemental profile of bone can be used even if they are not complete, making XRF analysis a more useful tool for forensic application.

### Limitation

In this study, we examined differences in the percentage of each element found in a scan, like a fingerprint, to describe comparative distributions of elements in calcified tissues. Actual concentrations were not calculated because the actual probing volume of each element was not known. Strongly different atomic numbers mean different efficiencies of excitation and detection, contributing to the variation of probing volumes of each element [52]. For the same element, different matrixes (e.g. soil, alloy and hydroxyapatite) can vary in probing volumes depending on the depth of X-ray penetration in the sample [53, 54]. Thus, to develop and calibrate a standard curve for each element, the actual probe volume in the hydroxyapatite matrix (bone, teeth and antler) is needed. However, our main objective was to do a comparative study to investigate the accumulation of elements across different species in organic tissue types. Thus, for this purpose we did not need actual concentrations of each element because the resulting semi-quantitative data were sufficient, similar to other investigations using a variety of sample types, such as bone [12, 17], teeth [12, 17], tusk [10] and fruit [55].

## Conclusion

This study showed the capacity of a handheld X-ray fluorescence (XRF) for scanning the elemental composition of biological samples including horns, antlers, teeth and bones (humerus) of various animal species. The data demonstrated the distribution of elements can be used to explain biological processes. For example, the high level of Fe in the elephant’s humerus bone suggests that the hemopoietic system may be distributed inside the entire long bone as they lack a marrow cavity. The XRF technique also can serve as a tool for species classification by the elemental profile of horn, antler, teeth and bone, with scans of the humerus bone being the most effective. Our results demonstrated that a handheld XRF was an effective tool for biological and forensic investigation.

## Ethical Approval

No ethical approval was required for this study.

## Declaration of Interest

The authors report no conflicts of interest. The authors alone are responsible for the content and writing of the paper.

## Supporting Information

S1 File Specimen numbers list. The list and number of skull, teeth and humerus specimens were used in this study
(DOCX)
