# Supplementary material for: Elemental Analysis of Bone, Teeth, Horn and Antler in Different Animal Species Using Non-Invasive Handheld X-Ray Fluorescence
Source: PLoS One. 2016 May 19;11(5):e0155458. doi: 10.1371/journal.pone.0155458 (PMC4873253; doi:10.1371/journal.pone.0155458)
Supplement: S1 File — The list and number of skull, teeth and humerus specimens were used in this study (DOCX) [file pone.0155458.s001.docx]

**Specimen numbers list**

**Skull samples**

1. Buffalo (Asiatic- *Bubalus bubalis*; n=3), specimen number; Buf-01, Buf-02, Buf-03
2. Barbary sheep (*Ammotragus lervia*; n=1), specimen number; She-Bar-01
3. Domestic goat (*Capra hircus*; n=3), specimen number; Goa-01, Goa-02, Goa-03
4. Eland (*Tragelaphus oryx;* n=1), specimen number; Ela-01
5. Gemsbuck (*Oryx gazella*; n=1), specimen number; Gem-01
6. Grant's gazelle (*Nanger granti*; n=2), specimen number; Gra-01, Gra-02
7. Greater kudu (*Tragelaphus strepsiceros*; n=1), specimen number; Gre-01
8. Nyala (*Tragelaphus angasii*; n=1), specimen number; Nya-01
9. Red lechwe (*Kobus leche;* n=1), specimen number; Red-01
10. Sitatunga (*Tragelaphus spekii*; n=1), specimen number; Sit-01
11. Spotted deer (*Axis axis*; n=2), specimen number; Spo-01, Spo-02
12. Sunda sambar (*Rusa timorensis;* n=2), specimen number; Sun-01, Sun-02

**Teeth sample**

1. Deer (*Odocoileus virginianus*, n=3), specimen number; Dee-01, Dee-02, Dee-03
2. Dog (*Canis lupus familiaris*, n=5), specimen number; V64, V67, V73, V74, V75
3. Elephant (Asian-; *Elephas maximus*, n=2); specimen number; Ele-01, Ele -02,
4. Horse (*Equus ferus caballus*, n=3), specimen number; Hor-01, Hor-02, Hor-03
5. Human (*Homo sapiens*, n=5), specimen number; E-G1, E-G2, E-G3, E-G4, E-H3
6. Monkey (Assam macaques; *Macaca assamensis*, n=5), specimen number; Mon-01, Mon-02, Mon-03, Mon-04, Mon-05
7. Dolphins (Spinner-; *Stenella longirostris*, n=2), specimen number; Dol-01, Dol-02
8. Crocodile (*Crocodylus siamensis*, n=2), specimen number; Cro-01, Cro-02

**Humerus sample**

1. Buffalo (Asiatic-; *Bubalus bubalis*; n=6), specimen number; Buf-01 (humerus right and left), Buf-02 (humerus right and left), Buf-03 (humerus right and left)
2. Cat (*Felis catus*; n=8), specimen number; Cat-01 (humerus right and left), Cat -02 (humerus right and left), Cat-03 (humerus right and left), Cat-04 (humerus right and left)
3. Dog (*Canis lupus familiaris*; n=10), V64(humerus right and left), V67(humerus right and left), V73(humerus right and left), V74(humerus right and left), V75 (humerus right and left)
4. Dolphin (Spinner-, *Stenella longirostris*; n=4), specimen number; Dol-01 (humerus right and left), Dol-02 (humerus right and left)
5. Elephant (Asian-, *Elephas maximus*; n=6), specimen number; Ele-01 (humerus right and left), Ele-02 (humerus right and left), Ele-03 (humerus right and left)
6. Horse (*Equus ferus caballus*; n=6), specimen number; Hor-01 (humerus right and left), Hor-02 (humerus right and left), Hor-03 (humerus right and left)
7. Human (*Homo sapiens*; n=10), specimen number; E-G1(humerus right and left), E-G2 (humerus right and left), E-G3 (humerus right and left), E-G4 (humerus right and left), E-H3 (humerus right and left)
8. Hyena (*Hyaena hyaena;* n=4), specimen number; Hye-01 (humerus right and left), Hye-02 (humerus right and left)
9. Lion (*Panthera leo*; n=2), specimen number; Lio-01 (humerus right and left)
10. Malayan tapir (*Tapirus indicus*; n=2), specimen number; Mal-01 (humerus right and left)
11. Monkey (Assam Macaques, *Macaca assamensis*; n=10), specimen number; Mon-01 (humerus right and left), Mon-02 (humerus right and left), Mon-03 (humerus right and left), Mon-04 (humerus right and left), Mon-05 (humerus right and left)
12. Pig (*Sus scrofa domesticus;* n=6), specimen number; Pig-01 (humerus right and left), Pig-02 (humerus right and left), Pig-03 (humerus right and left)
13. Sheep (*Ovis aries;* n=6), specimen number; She-01 (humerus right and left), She-02 (humerus right and left), She-03 (humerus right and left)
14. Tiger (*Panthera tigris*; n=2), specimen number; Tig-01 (humerus right and left), Tig-02 (humerus right and left)
